# Glutathione peroxidase 2 expression in human tumors: a tissue microarray study on 18,555 tumors

**DOI:** 10.3389/fonc.2026.1809848

**Published:** 2026-05-04

**Authors:** Viktoria Chirico, Niklas Jahn, Seyma Büyücek, Nina Schraps, Maximilian Lennartz, Katharina Möller, Elena Bady, Lisa Hornsteiner, Henning Plage, Claudia Hube-Magg, Martina Kluth, Frank Jacobsen, Florian Viehweger, David Dum, Andrea Hinsch, Christoph Fraune, Christian Bernreuther, Patrick Lebok, Guido Sauter, Till S Clauditz, Till Krech, Andreas H Marx, Ronald Simon, Eike Burandt, Natalia Gorbokon, Sarah Minner, Stefan Steurer

**Affiliations:** 1 Institute of Pathology, University Medical Center Hamburg-Eppendorf, Hamburg, Germany; 2 General, Visceral and Thoracic Surgery Department and Clinic, University Medical Center Hamburg-Eppendorf, Hamburg, Germany; 3 Department of Urology, Charité Berlin, Berlin, Germany; 4 Institute of Pathology, Clinical Center Osnabrueck, Osnabrueck, Germany; 5 Department of Pathology, Academic Hospital Fuerth, Fuerth, Germany

**Keywords:** glutathione peroxidase 2, GPX2, human carcinomas, immunohistochemistry, tissue microarray

## Abstract

**Background:**

Glutathione peroxidase 2 (GPX2) has a pivotal role in removing reactive oxygen species (ROS) from cells. Although the number of studies analyzing GPX2 in cancer is still limited, data suggesting a role of altered GPX2 expression in various cancer types are accumulatin.

**Methods:**

To better comprehend the role of GPX2 expression in cancer, GPX2 was analyzed by immunohistochemistry on tissue microarrays containing 18,555 samples from 148 different tumor types.

**Results:**

A total of 95 of 148 tumor categories showed GPX2 expression in at least one case, and 61 tumor categories contained at least one strongly positive case. GPX2 positivity was most seen in colorectal adenocarcinomas (97.9%) and adenomas (100%), non-invasive urothelial carcinomas (88.9-100%), pancreatico-biliary cancers (83.4-94.7%), Brenner tumors of the ovary (89.7%), gastro-esophageal adenocarcinomas (83.1-87.3%), and seminomas (85.6%). Clinically important cancer types with infrequent and often weak GPX2 staining included sarcomas, lymphomas, high-grade serous ovarian carcinomas, prostatic adenocarcinomas, melanomas, mesotheliomas, and renal cell carcinomas. Reduced GPX2 staining was linked to microsatellite instability (MSI, p<0.0001), advanced pT stage (p=0.0044), nodal metastasis (p=0.0093), V1 (p=0.0020) and L1 (p=0.0057) in colorectal adenocarcinoma, invasive growth (p<0.0001), high grade (p=0.0013), nodal metastasis (p=0.0005), V1 (p=0.0004) and L1 (p=0.0001) in urothelial carcinoma, high grade in pancreatic ductal adenocarcinoma (p=0.0233), and MSI in gastric adenocarcinoma (p=0.0325).

**Conclusion:**

It is concluded, that GPX2 expression is common in cancer and preferably occurs in tumor entities derived from normal cell types with high GPX2 expression. In these tumors, reduced GPX2 expression is often linked to features of aggressive disease.

## 1 Introduction

Glutathione peroxidase 2 (GPX2) is one out of eight known glutathione peroxidases (GPX1-8) in humans (1, 2). It plays a major role in removing potentially damaging reactive oxygen species (ROS) from cells by catalyzing the reduction of hydrogen peroxide to water (1). Among normal tissues, highest GPX2 levels have been found in the gastrointestinal tract, including the liver, stomach, small intestine, and colon (2, 3). In mice, GPX2 mRNA expression reacts to changes in the luminal microflora (4). It was therefore suggested that glutathione peroxidases play a role in the prevention of GI tract inflammation (4, 5). GPX2 knock-out mice are subject to an increased apoptosis rate at the colonic crypt bases (6). As GPX2 expression is highest in the crypt grounds and progressively declining towards the luminal surface (7), it is thought to play a role in cell proliferation and self-renewal of the intestinal mucosa (8).

The number of studies analyzing GPX2 in cancer is still limited but data suggesting a role of altered GPX2 expression in various cancer types are accumulating. In urinary bladder cancer, data mining from a published transcriptomic database had identified GPX2 as the most significantly downregulated gene among those which respond to oxidative stress (9) and immunohistochemical studies found associations between low GPX2 expression and invasive tumor growth and poor patient outcome (9, 10). In nasopharyngeal carcinoma, Liu et al (11) found a higher GPX2 expression than in normal nasopharyngeal tissues and high GPX2 expression was linked to advanced stage in cancer patients. GPX2 was also identified as a useful component of a prognostic gene signature in pulmonary adenocarcinoma (12). In castration-resistant prostate cancer, patients with high GPX2 expression in biopsy specimen had a significantly shorter prostate-specific antigen recurrence-free survival and overall survival than those with low GPX2 expression (13). In hepatocellular carcinoma (HCC), GPX2 levels markedly affected lenvatinib-induced ROS levels and apoptosis in tumor cells and also predicted response to lenvatinib therapy in clinical patients (14). However, many other cancer types have so far not been analyzed for GPX2 expression and its potential clinical role.

To better understand the prevalence and significance of GPX2 expression in cancer, a comprehensive study analyzing a large number of neoplastic and non-neoplastic tissues under highly standardized conditions is needed. Therefore, GPX2 expression was analyzed in more than 15,000 tumor tissue samples from 148 different tumor types and subtypes as well as 76 non-neoplastic tissue categories by immunohistochemistry (IHC) in a tissue microarray (TMA) format in this study.

## 2 Materials and methods

### 2.1 Tissue microarrays

The normal tissue TMA was composed of 8 samples from 8 different donors for each of 76 different normal tissue types (608 samples on one slide). The cancer TMAs contained a total of 18,555 primary tumors from 148 tumor types and subtypes. Detailed histopathological data on grade, pathological tumor stage (pT) or pathological lymph node status (pN) were available from 598 pancreatic cancers, 2,351 colorectal adenocarcinomas (CRC), 327 gastric adenocarcinomas, 518 thyroid cancers, 40 endometrial ovarian cancers, 182 endometrioid endometrial carcinomas, 568 urothelial carcinomas, and 565 testicular seminomas. The composition of both normal and cancer TMAs is described in detail in the results section. All samples were from the archives of the Institutes of Pathology of the University Medical Center Hamburg Eppendorf, the Clinical Center Osnabrueck, and the Academic Hospital Fuerth in Germany. Tissues were fixed in 4% buffered formalin and then embedded in paraffin. The TMA manufacturing process was described earlier in detail (15, 16). In brief, one tissue spot (diameter: 0.6 mm) was transmitted from a cancer containing donor block into an empty recipient paraffin block. The use of archived remnants of diagnostic tissues for manufacturing of TMAs and their analysis for research purposes as well as patient data analysis has been approved by local laws (HmbKHG, §12) and by the local ethics committee (Ethics commission Hamburg, WF-049/09). All work has been carried out in compliance with the Helsinki Declaration.

### 2.2 Immunohistochemistry

Freshly cut TMA sections were immunostained on one day and in one experiment. Slides were deparaffinized with xylol, rehydrated through a graded alcohol series and exposed to heat-induced antigen retrieval for 5 minutes in an autoclave at 121 °C in pH 7.8 Tris-EDTA-Citrat (TEC) puffer. Endogenous peroxidase activity was blocked with Dako REAL Peroxidase-Blocking Solution (Agilent Technologies, Santa Clara, CA, USA; #S2023) for 10 minutes. Primary antibody specific against GPX2 protein (rabbit recombinant monoclonal, HMV301, ardoci GmbH, Hamburg, Germany) was applied at 37 °C for 60 minutes at a dilution of 1:150. For the purpose of antibody validation, the normal tissue TMA was also analyzed by the rabbit polyclonal GPX2 antibody (ab137431, abcam, Cambridge, UK) at a dilution of 1:1200 and an otherwise identical protocol. Bound antibody was then visualized using the EnVision Kit (Agilent, CA, USA; #K5007) according to the manufacturer’s directions. The sections were counterstained with haemalaun. For tumor tissues, the percentage of GPX2 positive tumor cells was estimated, and the staining intensity was semi-quantitatively recorded (0, 1+, 2+, 3+). For statistical analyses, the staining results were categorized into four groups as follows: Negative: no staining at all, weak staining: staining intensity of 1+ in ≤ 70% or staining intensity of 2+ in ≤ 30% of tumor cells, moderate staining: staining intensity of 1+ in > 70%, staining intensity of 2+ in > 30% but in ≤ 70% or staining intensity of 3+ in ≤ 30% of tumor cells, strong staining: staining intensity of 2+ in > 70% or staining intensity of 3+ in > 30% of tumor cells.

### 2.3 Statistics

Statistical calculations were performed with JMP18^®^ software (SAS^®^, Cary, NC, USA). Contingency tables and the chi²-test were performed to search for associations between GPX2 immunostaining and tumor phenotype. p-values <0.05 were considered as significant.

## 3 Results

### 3.1 Technical issues

A total of 15,654 (84.4%) of 18,555 tumor samples were interpretable in the TMA analysis. The remaining 2,901 (15.6%) samples were not analyzable due to a lack of unequivocal tumor cells or a lack of entire tissue spots. On the normal tissue TMA ≥4 samples were always interpretable per tissue type to determine GPX2 expression.

### 3.2 GPX2 immunostaining in normal tissues

**Figure 1:**
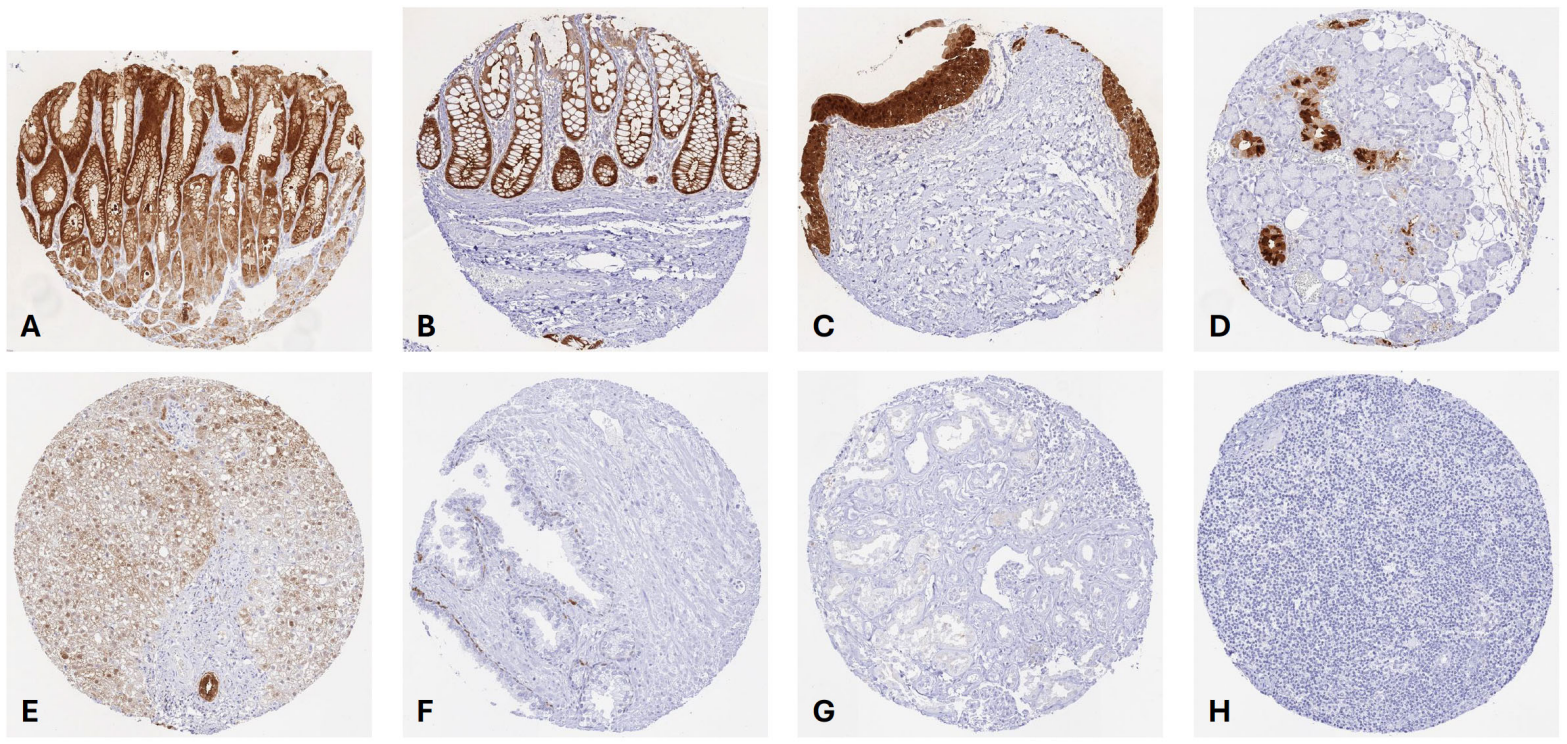
GPX2 staining in normal tissues. The panels show a strong predominantly cytoplasmic and nuclear GPX2 staining of the surface epithelium of the stomach **(A)**, epithelial cells of the colon **(B)**, urothelium of the urinary bladder **(C)** excretory ducts of the submandibular gland **(D)**, and a bile duct of the liver while hepatocytes only stain weakly **(E)**. A weak GPX2 staining also occurs in basal cells of prostate **(F)** while GPX2 staining is absent in the kidney **(G)** and in lymph nodes **(H)**.

GPX2 staining was most intense in the stomach (strongest staining of luminal epithelial cells and of parietal cells, only weak staining of other gastric gland cells), small intestine, and colorectum (strongest staining of basal crypt cells), excretory duct cells of the pancreas, gall bladder and bile duct epithelial cells, a subset of cells of excretory ducts of salivary glands, and urothelial cells (exception: staining was markedly less intense in umbrella cells). A variable, weak to strong GPX2 staining occurred in squamous epithelium of various sites (predominantly in basal and suprabasal cell layers), peripheral germinative cells of sebaceous glands, basal cells of the prostate, basal cells of the caput epididymis, and respiratory epithelium. GPX2 staining was weak to moderate in hepatocytes. GPX2 immunostaining was usually cytoplasmic and nuclear but the apical membranes of gastrointestinal epithelial cells did also show staining. Representative images are shown in **Figure 1**. GPX2 staining was absent in heart muscle, skeletal muscle, smooth muscle, myometrium, brain, pituitary gland, thyroid, parathyroid, adrenal gland, lung, kidney, testis, breast, endocervix, endometrium, fallopian tube, ovary, placenta, endothelial cells, fat, bone marrow, lymph node, spleen, and the thymus. All GPX2 positive cell types detected by HMV301 were also confirmed by using ab137431. For ab137431 an additional nuclear staining was seen in several tissues, including for example pneumocytes, chorion cells and trophoblastic cells of the placenta, and epithelial cells and pituicytes of the hypophysis (**Supplementary Figure 1**).

### 3.3 GPX2 immunostaining in neoplastic tissues

**Figure 2:**
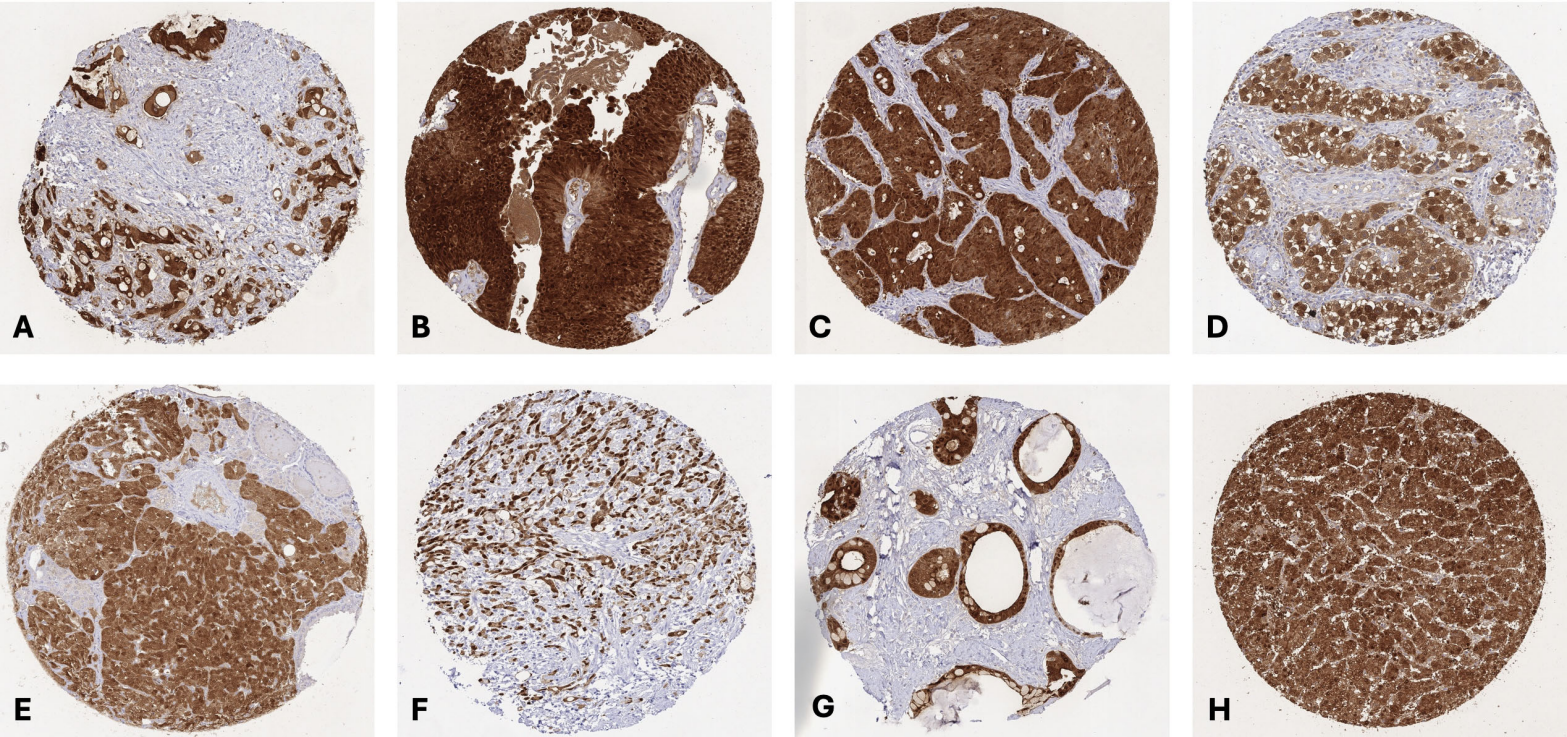
GPX2 immunostaining in cancer. The panels show a strong GPX2 staining in a ductal adenocarcinoma of the pancreas **(A)**, a non-invasive high grade papillary urothelial carcinoma **(B)**, a colorectal adenocarcinoma **(C)**, a seminoma of the testis **(D)**, a medullary carcinoma of the thyroid **(E)**, a gastric adenocarcinoma **(F)**, a mucinous carcinoma of the ovary **(G)**, and a hepatocellular carcinoma **(H)**.

**Figure 3:**
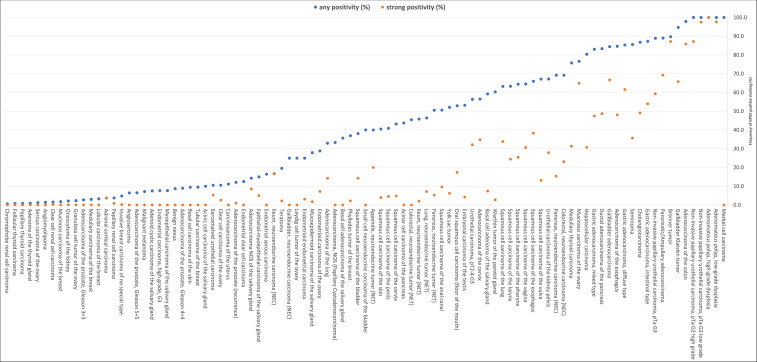
Ranking order of human tumors according to GPX2 immunostaining. Orange dots show the percentage of strongly stained samples, while blue dots show the percentage of positive samples of any intensity.

Detectable GPX2 staining occurred in 5,919 (37.8%) of the 15,654 analyzable tumors, including 1,177 (7.5%) with weak, 1,171 (7.5%) with moderate, and 3,571 (22.8%) with strong positivity. A total of 95 of 148 (64.2%) tumor categories showed GPX2 expression in at least one case, while 61 included at least one case with strong GPX2 positivity, and 39 showed GPX2 staining in ≥50% of cases (**Table 1**). GPX2 positivity was most seen in colorectal adenocarcinomas (97.9%) and adenomas (100%), non-invasive urothelial carcinomas (88.9-100%), pancreatico-biliary cancers (83.4-94.7%), Brenner tumors of the ovary (89.7%), gastro-esophageal adenocarcinomas (83.1-87.3%), seminomas (85.6%), and hepatocellular carcinomas (80.4%). Clinically important cancer types which often showed low or absent GPX2 staining included endometrial cancer (GPX2 positive in up to 25%), adenocarcinomas of the prostate (up to 12.1%), malignant melanoma (7.1%), sarcomas (up to 6.5% positive), renal cell carcinomas (up to 3.8%), high-grade serous ovarian carcinoma (1.2%), malignant lymphomas (0.0%) and malignant mesothelioma (0.0%). Representative images are shown in **Figure 2**. A graphical representation of a ranking order of GPX2 positive and strongly positive cancers is given in **Figure 3**. The relationship between GPX2 staining and histopathological, molecular, and clinical parameters are summarized in **Table 2**. Reduced GPX2 staining was linked to microsatellite instability (MSI, p<0.0001), advanced pT stage (p=0.0044), nodal metastasis (p=0.0093), V1 (p=0.002) and L1 status (p=0.0057) in colorectal adenocarcinoma, V1 (p=0.0095) and L1 status (p=0.0038) in microsatellite stable CRCs, invasive disease (p<0.0001) in urothelial carcinoma, high grade (p=0.0013), nodal metastasis (p=0.0005), V1 (p=0.0004) and L1 status (p=0.0001) in muscle-invasive urothelial carcinoma, high grade in pancreatic ductal adenocarcinoma (p=0.0233), and MSI in gastric adenocarcinoma (p=0.0325). The GPX2 expression level was unrelated to features of cancer aggressiveness in endometrioid ovarian carcinoma, endometrial carcinoma, hepatocellular carcinoma, as well as in follicular and papillary thyroid cancer.

**Table 1:** GPX2 immunostaining in human tumors.

Tumor classification			GPX2 immunostaining				
	Tumor entity	On TMA (n)	Analyzable (n)	Negative (%)	Weak (%)	Moderate (%)	Strong (%)
Tumors of the skin	Basal cell carcinoma of the skin	89	74	90.5	8.1	1.4	0.0
	Benign nevus	29	23	91.3	8.7	0.0	0.0
	Squamous cell carcinoma of the skin	145	126	59.5	24.6	11.9	4.0
	Malignant melanoma	65	56	92.9	3.6	3.6	0.0
	Malignant melanoma lymph node metastasis	86	86	100.0	0.0	0.0	0.0
	Merkel cell carcinoma	2	1	0.0	0.0	100.0	0.0
Tumors of the head and neck	Squamous cell carcinoma of the larynx	109	90	36.7	23.3	15.6	24.4
	Squamous cell carcinoma of the pharynx	60	59	35.6	18.6	20.3	25.4
	Oral squamous cell carcinoma (floor of the mouth)	130	121	47.1	24.8	10.7	17.4
	Pleomorphic adenoma of the parotid gland	50	39	100.0	0.0	0.0	0.0
	Warthin tumor of the parotid gland	104	73	39.7	41.1	16.4	2.7
	Adenocarcinoma, NOS (Papillary Cystadenocarcinoma)	14	6	66.7	0.0	33.3	0.0
	Acinic cell carcinoma of the salivary gland	181	50	90.0	6.0	4.0	0.0
	Adenocarcinoma NOS of the salivary gland	109	35	85.7	2.9	2.9	8.6
	Adenoid cystic carcinoma of the salivary gland	180	40	92.5	5.0	2.5	0.0
	Basal cell adenocarcinoma of the salivary gland	25	14	64.3	28.6	7.1	0.0
	Basal cell adenoma of the salivary gland	101	27	40.7	37.0	14.8	7.4
	Epithelial-myoepithelial carcinoma of the salivary gland	53	20	85.0	5.0	5.0	5.0
	Mucoepidermoid carcinoma of the salivary gland	343	172	72.1	20.3	5.8	1.7
	Myoepithelial carcinoma of the salivary gland	21	13	92.3	0.0	7.7	0.0
	Myoepithelioma of the salivary gland	11	8	100.0	0.0	0.0	0.0
	Oncocytic carcinoma of the salivary gland	12	2	100.0	0.0	0.0	0.0
	Polymorphous adenocarcinoma, low grade, of the salivary gland	41	1	100.0	0.0	0.0	0.0
	Pleomorphic adenoma of the salivary gland	53	20	100.0	0.0	0.0	0.0
Tumors of the lung, pleura and thymus	Adenocarcinoma of the lung	196	176	67.0	11.4	7.4	14.2
	Squamous cell carcinoma of the lung	80	68	36.8	14.7	14.7	33.8
	Mesothelioma, epithelioid	40	31	100.0	0.0	0.0	0.0
	Mesothelioma, biphasic	29	21	100.0	0.0	0.0	0.0
	Thymoma	29	25	100.0	0.0	0.0	0.0
	Lung, neuroendocrine tumor (NET)	29	28	53.6	32.1	7.1	7.1
Tumors of the female genital tract	Squamous cell carcinoma of the vagina	78	62	35.5	16.1	17.7	30.6
	Squamous cell carcinoma of the vulva	157	137	32.8	25.5	28.5	13.1
	Squamous cell carcinoma of the cervix	136	125	56.8	23.2	15.2	4.8
	Adenocarcinoma of the cervix	23	23	43.5	4.3	17.4	34.8
	Endometrioid endometrial carcinoma	338	320	75.0	14.7	7.2	3.1
	Endometrial serous carcinoma	86	73	83.6	11.0	5.5	0.0
	Carcinosarcoma of the uterus	57	54	88.9	11.1	0.0	0.0
	Endometrial carcinoma, high grade, G3	13	13	92.3	0.0	7.7	0.0
	Endometrial clear cell carcinoma	9	8	87.5	12.5	0.0	0.0
	Endometrioid carcinoma of the ovary	130	111	71.2	8.1	13.5	7.2
	Serous carcinoma of the ovary	580	487	98.8	0.8	0.4	0.0
	Mucinous carcinoma of the ovary	101	77	23.4	5.2	6.5	64.9
	Clear cell carcinoma of the ovary	51	38	89.5	7.9	0.0	2.6
	Carcinosarcoma of the ovary	47	44	100.0	0.0	0.0	0.0
	Granulosa cell tumor of the ovary	44	43	97.7	2.3	0.0	0.0
	Leydig cell tumor of the ovary	4	4	75.0	25.0	0.0	0.0
	Sertoli cell tumor of the ovary	1	1	100.0	0.0	0.0	0.0
	Sertoli Leydig cell tumor of the ovary	3	3	100.0	0.0	0.0	0.0
	Steroid cell tumor of the ovary	3	3	100.0	0.0	0.0	0.0
	Brenner tumor	41	39	10.3	0.0	2.6	87.2
Tumors of the breast	Invasive breast carcinoma of no special type	1764	1636	95.2	3.7	0.9	0.2
	Lobular carcinoma of the breast	363	334	96.7	2.1	1.2	0.0
	Medullary carcinoma of the breast	34	33	97.0	3.0	0.0	0.0
	Tubular carcinoma of the breast	29	21	90.5	9.5	0.0	0.0
	Mucinous carcinoma of the breast	65	56	98.2	1.8	0.0	0.0
	Phyllodes tumor of the breast	50	46	63.0	13.0	21.7	2.2
Tumors of the digestive system	Adenomatous polyp, low-grade dysplasia	50	42	0.0	0.0	2.4	97.6
	Adenomatous polyp, high-grade dysplasia	50	49	0.0	0.0	0.0	100.0
	Adenocarcinoma of the colon	2483	2007	2.1	2.6	9.4	85.8
	Gastric adenocarcinoma, diffuse type	215	177	14.7	7.9	15.8	61.6
	Gastric adenocarcinoma, intestinal type	215	189	12.7	15.9	17.5	54.0
	Gastric adenocarcinoma, mixed type	62	59	16.9	10.2	25.4	47.5
	Adenocarcinoma of the esophagus	83	52	15.4	15.4	21.2	48.1
	Squamous cell carcinoma of the esophagus	76	47	34.0	19.1	8.5	38.3
	Squamous cell carcinoma of the anal canal	91	83	49.4	26.5	14.5	9.6
	Cholangiocarcinoma	58	53	13.2	20.8	17.0	49.1
	Gallbladder adenocarcinoma	51	45	15.6	6.7	11.1	66.7
	Gallbladder Klatskin tumor	42	38	5.3	15.8	13.2	65.8
	Hepatocellular carcinoma	312	306	19.6	26.1	23.5	30.7
	Ductal adenocarcinoma of the pancreas	659	577	16.6	13.3	21.3	48.7
	Pancreatic/Ampullary adenocarcinoma	98	91	11.0	9.9	9.9	69.2
	Acinar cell carcinoma of the pancreas	18	16	56.3	37.5	6.3	0.0
	Gastrointestinal stromal tumor (GIST)	62	56	100.0	0.0	0.0	0.0
	Appendix, neuroendocrine tumor (NET)	25	20	60.0	5.0	15.0	20.0
	Colorectal, neuroendocrine tumor (NET)	12	11	54.5	45.5	0.0	0.0
	Ileum, neuroendocrine tumor (NET)	53	48	54.2	31.3	12.5	2.1
	Pancreas, neuroendocrine tumor (NET)	101	95	49.5	27.4	17.9	5.3
	Colorectal, neuroendocrine carcinoma (NEC)	14	13	30.8	30.8	15.4	23.1
	Ileum, neuroendocrine carcinoma (NEC)	8	6	83.3	0.0	0.0	16.7
	Gallbladder, neuroendocrine carcinoma (NEC)	4	4	75.0	25.0	0.0	0.0
	Pancreas, neuroendocrine carcinoma (NEC)	14	13	30.8	23.1	30.8	15.4
Tumors of the urinary system	Non-invasive papillary urothelial carcinoma, pTa G2 low grade	87	80	0.0	0.0	2.5	97.5
	Non-invasive papillary urothelial carcinoma, pTa G2 high grade	80	70	0.0	8.6	4.3	87.1
	Non-invasive papillary urothelial carcinoma, pTa G3	126	108	11.1	13.9	15.7	59.3
	Urothelial carcinoma, pT2–4 G3	735	644	43.6	12.1	12.1	32.1
	Squamous cell carcinoma of the bladder	22	21	61.9	14.3	9.5	14.3
	Small cell neuroendocrine carcinoma of the bladder	5	5	60.0	20.0	20.0	0.0
	Sarcomatoid urothelial carcinoma	25	19	89.5	0.0	5.3	5.3
	Urothelial carcinoma of the kidney pelvis	62	61	32.8	19.7	19.7	27.9
	Clear cell renal cell carcinoma	1287	1213	98.4	0.9	0.4	0.2
	Papillary renal cell carcinoma	368	343	96.2	2.3	0.6	0.9
	Clear cell (tubulo) papillary renal cell carcinoma	26	22	100.0	0.0	0.0	0.0
	Chromophobe renal cell carcinoma	170	163	99.4	0.6	0.0	0.0
	Oncocytoma of the kidney	257	234	97.9	2.1	0.0	0.0
Tumors of the male genital organs	Adenocarcinoma of the prostate, Gleason 3 + 3	83	73	97.3	2.7	0.0	0.0
	Adenocarcinoma of the prostate, Gleason 4 + 4	80	67	91.0	6.0	3.0	0.0
	Adenocarcinoma of the prostate, Gleason 5 + 5	85	77	93.5	5.2	1.3	0.0
	Adenocarcinoma of the prostate (recurrence)	258	199	87.9	7.5	3.5	1.0
	Small cell neuroendocrine carcinoma of the prostate	2	2	100.0	0.0	0.0	0.0
	Seminoma	682	479	14.4	14.8	35.1	35.7
	Embryonal carcinoma of the testis	54	47	46.8	34.0	14.9	4.3
	Leydig cell tumor of the testis	31	31	100.0	0.0	0.0	0.0
	Sertoli cell tumor of the testis	2	2	100.0	0.0	0.0	0.0
	Sex cord stromal tumor of the testis	1	1	100.0	0.0	0.0	0.0
	Spermatocytic tumor of the testis	1	1	100.0	0.0	0.0	0.0
	Yolk sac tumor	53	48	47.9	39.6	6.3	6.3
	Teratoma	53	46	80.4	13.0	4.3	2.2
	Squamous cell carcinoma of the penis	92	88	59.1	27.3	9.1	4.5
Tumors of endocrine organs	Adenoma of the thyroid gland	113	104	99.0	1.0	0.0	0.0
	Papillary thyroid carcinoma	391	355	99.2	0.3	0.6	0.0
	Follicular thyroid carcinoma	154	126	99.2	0.8	0.0	0.0
	Medullary thyroid carcinoma	111	99	24.2	16.2	28.3	31.3
	Parathyroid gland adenoma	43	43	100.0	0.0	0.0	0.0
	Anaplastic thyroid carcinoma	45	42	100.0	0.0	0.0	0.0
	Adrenal cortical adenoma	48	48	100.0	0.0	0.0	0.0
	Adrenal cortical carcinoma	27	27	96.3	0.0	0.0	3.7
	Pheochromocytoma	51	50	100.0	0.0	0.0	0.0
Tumors of hematopoetic and lymphoid tissues	Hodgkin’s lymphoma	103	86	100.0	0.0	0.0	0.0
	Small lymphocytic lymphoma, B-cell type (B-SLL/B-CLL)	50	50	100.0	0.0	0.0	0.0
	Diffuse large B cell lymphoma (DLBCL)	113	113	100.0	0.0	0.0	0.0
	Follicular lymphoma	88	88	100.0	0.0	0.0	0.0
	T-cell non-Hodgkin’s lymphoma	25	25	100.0	0.0	0.0	0.0
	Mantle cell lymphoma	18	18	100.0	0.0	0.0	0.0
	Marginal zone lymphoma	16	16	100.0	0.0	0.0	0.0
	Diffuse large B-cell lymphoma (DLBCL) in the testis	16	16	100.0	0.0	0.0	0.0
	Burkitt lymphoma	5	5	100.0	0.0	0.0	0.0
Tumors of soft tissue and bone	Granular cell tumor	23	16	100.0	0.0	0.0	0.0
	Leiomyoma	50	47	100.0	0.0	0.0	0.0
	Leiomyosarcoma	94	82	100.0	0.0	0.0	0.0
	Liposarcoma	96	81	100.0	0.0	0.0	0.0
	Malignant peripheral nerve sheath tumor (MPNST)	15	13	100.0	0.0	0.0	0.0
	Myofibrosarcoma	26	25	100.0	0.0	0.0	0.0
	Angiosarcoma	42	31	93.5	6.5	0.0	0.0
	Angiomyolipoma	91	69	98.6	1.4	0.0	0.0
	Dermatofibrosarcoma protuberans	21	16	100.0	0.0	0.0	0.0
	Ganglioneuroma	14	13	100.0	0.0	0.0	0.0
	Kaposi sarcoma	8	5	100.0	0.0	0.0	0.0
	Neurofibroma	117	112	100.0	0.0	0.0	0.0
	Sarcoma, not otherwise specified (NOS)	74	64	100.0	0.0	0.0	0.0
	Paraganglioma	41	39	100.0	0.0	0.0	0.0
	Ewing sarcoma	23	12	100.0	0.0	0.0	0.0
	Rhabdomyosarcoma	7	6	100.0	0.0	0.0	0.0
	Schwannoma	122	118	100.0	0.0	0.0	0.0
	Synovial sarcoma	12	10	100.0	0.0	0.0	0.0
	Osteosarcoma	19	13	100.0	0.0	0.0	0.0
	Chondrosarcoma	15	12	100.0	0.0	0.0	0.0
	Rhabdoid tumor	5	5	100.0	0.0	0.0	0.0
	Solitary fibrous tumor	17	17	100.0	0.0	0.0	0.0

**Table 2:** GPX2 immunostaining and tumor phenotype.

Tumor entity	Pathological parameters		GPX2 immunostaining				
		n	Negative (%)	Weak (%)	Moderate (%)	Strong (%)	P
Hepatocellular carcinoma	pT1	78	21.8	20.5	29.5	28.2	0.3349
	pT2	85	15.3	23.5	21.2	40.0	
	pT3-4	62	17.7	32.3	17.7	32.3	
	pN0	76	13.2	25.0	25.0	36.8	0.2028
	pN+	43	16.2	14.0	16.3	53.5	
	G 1	37	29.8	21.6	21.6	27.0	0.0748
	G 2	132	14.4	22.0	24.2	39.4	
	G 3	53	24.4	34.0	20.8	20.8	
Urothelial bladder carcinoma	pTa G2 low	80	0.0	0.0	2.5	97.5	<0.0001
	pTa G2 high	70	0.0	8.6	4.3	87.1	
	pTa G3	78	9.0	15.4	16.6	59	
	pT2	136	46.3	10.3	9.6	33.8	0.1692
	pT3	233	45.9	15.0	8.6	30.5	
	pT4	109	35.8	11.0	16.5	36.7	
Urothelial bladder carcinoma, pT2-4	G2	25	12	8	16	64	0.0013
	G3	452	45.4	13.1	10.3	31.2	
	pN0	280	49.6	10.4	7.1	32.9	0.0005
	pN+	167	34.7	16.2	17.4	31.7	
	R0	362	45	11.9	9.7	33.4	0.5611
	R1	87	41.4	14.9	13.8	29.9	
	L0	196	53.6	11.7	7.1	27.6	0.0001
	L1	164	31.1	14	15.9	39	
	V0	261	49.8	13	9.2	28	0.0004
	V1	85	25.9	11.8	16.4	45.9	
Adenocarcinoma of the pancreas	pT1	13	7.7	15.4	15.4	61.5	0.2779
	pT2	62	9.7	19.4	29	41.9	
	pT3	357	16.0	14.0	20.1	49.9	
	pT4	27	18.5	3.7	14.8	63.0	
	G1	16	0.0	0.0	18.7	81.3	0.0233
	G2	321	14.6	15.9	22.1	47.4	
	G3	99	18.2	12.1	18.2	51.5	
	pN0	92	14.1	14.1	21.8	50.0	0.9967
	pN+	366	14.8	14.2	20.7	50.3	
	MMR proficient	421	15.4	13.3	21.4	49.9	0.4425
	MMR deficient	3	33.3	0.0	0.0	66.7	
Adenocarcinoma of the stomach	pT1-2	60	15.0	6.7	21.6	56.7	0.9219
	pT3	126	15.1	10.3	19.8	54.8	
	pT4	127	11.8	11.8	18.9	57.5	
	pN0	83	13.3	14.5	16.8	55.4	0.5787
	pN+	229	14.0	9.2	20.5	56.3	
	MMR proficient	261	10.7	11.5	20.7	57.1	0.0325
	MMR deficient	41	22.0	19.5	24.4	34.1	
Endometrioid endometrial carcinoma	pT1	112	74.1	15.2	7.1	3.6	0.8232
	pT2	24	58.3	25	12.5	4.2	
	pT3-4	37	67.6	16.2	10.8	5.4	
	pN0	50	58.0	16.0	16.0	10.0	0.0141
	pN+	30	83.4	13.3	0.0	3.3	
Endometrioid carcinoma of the ovary	pT1	26	38.5	11.5	23.1	26.9	0.5254
	pT2	4	0.0	50.0	50.0	0.0	
	pT3	6	66.6	16.7	0.0	16.7	
	pN0	19	47.4	21.1	26.2	5.3	0.2922
	pN1	6	66.6	16.7	0.0	16.7	
Adenocarcinoma of the colon	pT1	66	0.0	0.0	3.0	97.0	0.0044
	pT2	378	0.8	1.9	8.4	88.9	
	pT3	1067	2.4	2.7	9.5	85.4	
	pT4	380	3.4	3.9	11.1	81.6	
	pN0	1005	2.1	1.5	9.0	87.4	0.0093
	pN+	883	2.4	4.0	10.0	83.6	
	V0	1351	1.9	2.1	8.5	87.5	0.002
	V1	504	3.2	4.2	12.0	80.6	
	L0	983	2.1	2.2	9.4	86.3	0.3041
	L1	369	3.8	2.7	10.6	82.9	
	right side	369	3.8	2.7	10.6	82.9	0.3041
	left side	983	2.1	2.2	9.4	86.3	
	MMR proficient	967	1.9	1.8	7.6	88.7	<0.0001
	MMR deficient	72	6.9	8.3	19.5	65.3	
Adenocarcinoma of the colon, MMR proficient	pT1	36	0.0	0.0	2.8	97.2	0.1194
	pT2	203	0.5	0.5	6.9	92.1	
	pT3	529	2.5	2.5	7.3	87.7	
	pT4	190	2.1	1.6	10.0	86.3	
	pN0	502	1.8	1.2	6.8	90.2	0.3141
	pN+	450	2.0	2.4	8.7	86.9	
	V0	676	1.5	1.5	6.0	91.0	0.0095
	V1	265	3.0	2.3	11.7	83.0	
	L0	381	1.0	0.8	4.8	93.4	0.0038
	L1	549	2.2	2.4	9.2	86.2	
	right side	224	2.2	1.3	6.3	90.2	0.7262
	left side	737	1.8	1.9	8.0	88.3	
	RAS mutation	294	2.4	2.4	8.1	87.1	0.3179
	RAS mutation	234	2.1	0.9	5.5	91.5	
	BRAF wildtype	70	1.4	1.4	7.2	90.0	0.7974
	BRAF V600E mutation	5	0.0	0.0	0.0	100.0	
Follicular thyroid carcinoma	pT1	11	90.9	9.1	0.0	0.0	0.1288
	pT2	38	100.0	0.0	0.0	0.0	
	pT3-4	33	100.0	0.0	0.0	0.0	
	pN0	23	95.7	4.3	0.0	0.0	0.6798
	pN1	2	100.0	0.0	0.0	0.0	
Papillary thyroid carcinoma	pT1	139	100.0	0.0	0.0	0.0	0.1263
	pT2	75	100.0	0.0	0.0	0.0	
	pT3-4	94	96.8	1.1	2.1	0.0	
	pN0	85	98.8	0.0	1.2	0.0	0.8302
	pN1	115	99.1	0.0	0.9	0.0	
Seminoma	pT1	245	13.9	15.9	33.1	37.1	0.0521
	pT2	91	6.6	11.0	51.6	30.8	
	pT3	40	17.5	15.0	40.0	27.5	
	V0	306	13.1	16.3	35.0	35.6	0.0824
	V+	41	12.2	4.9	51.2	31.7	
	L0	271	13.3	16.6	33.2	36.9	0.1162
	L+	79	11.4	11.4	48.1	29.1	
	M0 at point of surgery	381	12.6	14.7	37.5	35.2	0.7355
	M+ at point of surgery	2	0.0	0.0	50.0	50.0	

G, grade; pN, pathologic lymph node status; pT, pathologic tumor stage; L, lymphatic invasion status; V, blood vessel invasion status; RAS, rat sarcoma virus; BRAF, v-RAF murine sarcoma viral oncogene homolog B1; MMR, mismatch repair.

## 4 Discussion

The successful analysis of 15,654 tumors from 148 tumor types and subtypes provides a comprehensive overview on GPX2 protein expression in human tumors. Based on the highly standardized TMA approach in which virtually all parameters that can influence the outcome of IHC studies such as antibody properties, staining protocol, slide age (time between cutting and staining of tissue slides), slide storage, section thickness, tissue quantity per patient, and the interpreter were largely identical for all tumors, we assume that our data properly describe the relative GPX2 expression levels in these tumor entities. Our data suggest that GPX2 expression predominates in cancer entities which are derived from cell types with high GPX2 expression levels. Tumor types with the highest GPX2 staining such as colorectal adenocarcinoma, non-invasive papillary urothelial carcinoma, carcinoma of the biliary tract, gastric adenocarcinoma, ductal adenocarcinoma of the pancreas, or hepatocellular carcinoma are derived from cell types with prominent GPX2 expression. Conversely, tumor entities derived from GPX2 negative cell types were mostly GPX2 negative such as for example sarcomas, lymphomas, prostatic adenocarcinomas, malignant melanomas, and renal cell carcinomas. Seminoma of the testis is a major exception regarding this observation. Cells of the spermatogenesis were always negative in normal testis but seminomas were GPX2 positive in >85% of cases. As GPX2 neo-expression or upregulation may play a significant role in testicular cancer development it needs to be tested whether GPX2 IHC might have diagnostic utility in the distinction of early neoplastic cells in testicular biopsies.

The availability of sufficiently large tumor cohorts from several different tumor entities enabled us to analyze the relationship between GPX2 expression and histopathological parameters of cancer aggressiveness as well as relevant molecular cancer features. Most data from our project suggest an association of reduced GPX2 expression with unfavorable tumor features. This may especially apply to cancer types derived from GPX2 expressing normal cells such as colorectal adenocarcinoma, urothelial carcinoma and ductal pancreatic adenocarcinoma. In line with our data, other studies examining GPX2 expression by IHC or RNA analysis have also found associations between low GPX2 levels and increased cancer aggressiveness in esophageal cancer (17), breast cancer (18), and urothelial carcinoma (9). Functional studies identified associations between reduced GPX2 expression and cancer associated cell properties such as migration (19), invasion (19), metabolic changes (18), angiogenesis (18), and pro-tumorigenic signaling through accumulation of ROS (18). In a study employing gene set enrichment analysis (GSEA), Ren et al. (18) found an enrichment of prooncogenic signaling pathways in breast cancer cells with low GPX2 expression. These authors speculated that increased ROS signaling in GPX2 deficient cancers had promoted angiogenesis, tumor growth and a metabolic shift from ATP production through oxidative phosphorylation to aerobic glycolysis. Banning et al. (19) found increased migration and invasion in a colon cancer cell line after GPX2 downregulation by siRNA, which was accompanied by reduced suppression of the tumor-promoting COX-2 and its metabolite PGE_2_ (20). A cancer protective role of GPX2 is well known in mice where the knockdown of GPX2 and GPX1 led to ileocolitis and intestinal cancer (5, 21, 22). As an alternative explanation for the association between low GPX2 expression and unfavorable tumor parameters, it cannot be excluded that a GPX2 expression loss can be caused by random alterations occurring during dedifferentiation of tumor cells which regularly takes place during tumor progression. The significant link between reduced GPX2 expression and MSI in colorectal and gastric adenocarcinomas is in line with recent results from Cui et al. (23) who identified GPX2 downregulation as an MSI-related gene alteration by using a series of publicly available databases.

It is of note that others have described significant associations between GPX2 overexpression and unfavorable tumor features (11, 24–27). These reports also include tumor entities such as colorectal cancer (28), gastric cancer (26), and hepatocellular carcinomas (27) where we found opposite results or could not confirm a relationship between GPX2 expression levels and aggressive disease. Mechanisms that have been suggested to cause aggressive cell behavior in case of GPX2 overexpression include a promotion of epithelial-mesenchymal transition (EMT) (25, 29), increased cell proliferation (29–31), migration (29–31), and invasion (25, 29), as well as antiapoptotic signaling (8, 32). Cell line studies have suggested that GPX2 can activate EMT through WNT/ß-catenin signaling (29–31). While GPX2 is required to maintain normal self-renewal of the gastrointestinal epithelium in healthy tissue and to suppress inflammatory processes, other functional effects such as inhibition of apoptosis have been suggested as a potential cause of cancer cell growth in cells with increased GPX2 expression (8). Previous studies have suggested that GPX2 induction by the transcription factor ΔNp63 may inhibit the apoptotic response to oxidative stress in tumor cells (8, 32). It is also of interest that high GPX2 levels in cancer have been suspected to be associated with CIS-platin resistance in a study on 152 lung cancers (24).

Given the large scale of our study, emphasis was placed on a thorough validation of our assay. The International Working Group for Antibody Validation (IWGAV) has proposed that antibody validation for IHC on formalin fixed tissues should include either a comparison with a different independent antibody or a comparison with expression data obtained by an independent second method (33). For the antibody HMV301 specific detection of GPX2 is suggested by the highest immunostaining levels in tissues with highest documented GPX2 RNA expression (stomach, duodenum, small intestine, appendix, colon, rectum, gallbladder, urinary bladder) and the absence of staining in most tissues lacking detectable RNA (thymus, tonsil, lymph node, spleen, bone marrow, skeletal and heart muscle, placenta, endocrine organs, endometrium, fallopian tube, ovary, breast, testis, brain) (34–37). The few tissues without documented GPX2 RNA expression but GPX2 positivity by HMV301 had either very few positive cells that were probably not detected in RNA studies (basal cells of the caput epididymis) or were previously not analyzed on the RNA level (respiratory epithelium). True expression of basal cells of the caput epididymis and respiratory epithelium as well as of other HMV301 positive cell types is corroborated by identical staining patterns obtained by ab137431. The additional staining of nuclei of various tissues such as pneumocytes, chorion cells and trophoblastic cells of the placenta as well as of epithelial cells and pituicytes of the hypophysis by ab137431 is suggestive of a cross-reactivity of ab137431 with one or several nuclear proteins occurring in these cells. This cross-reactivity further documents the independent nature of the validation antibody ab137431. The detection of this cross-reactivity demonstrates the power of antibody validation on 76 different normal tissues, representing a near complete set of human organs which are likely to contain a near complete spectrum of human proteins and their post-translational modifications, all of which have been evaluated for potential cross-reactivity.

In summary, our data provide a comprehensive overview on the prevalence and levels of GPX2 expression in cancer. The data show that GPX2 expression preferably occurs in tumor entities derived from normal cell types with high GPX2 expression. In these tumors, reduced GPX2 expression is often linked to features of aggressive disease.

## Data Availability

The original contributions presented in the study are included in the article/**Supplementary Material**. Further inquiries can be directed to the corresponding author.
