# Supplementary material for: Glutathione peroxidase 2 expression in human tumors: a tissue microarray study on 18,555 tumors
Source: Front Oncol. 2026 May 4;16:1809848. doi: 10.3389/fonc.2026.1809848 (PMC13180585; doi:10.3389/fonc.2026.1809848)
Supplement: Supplementary file 1 [file DataSheet1.docx]

Supplementary Material

# Supplementary Data

## Supplementary Figures


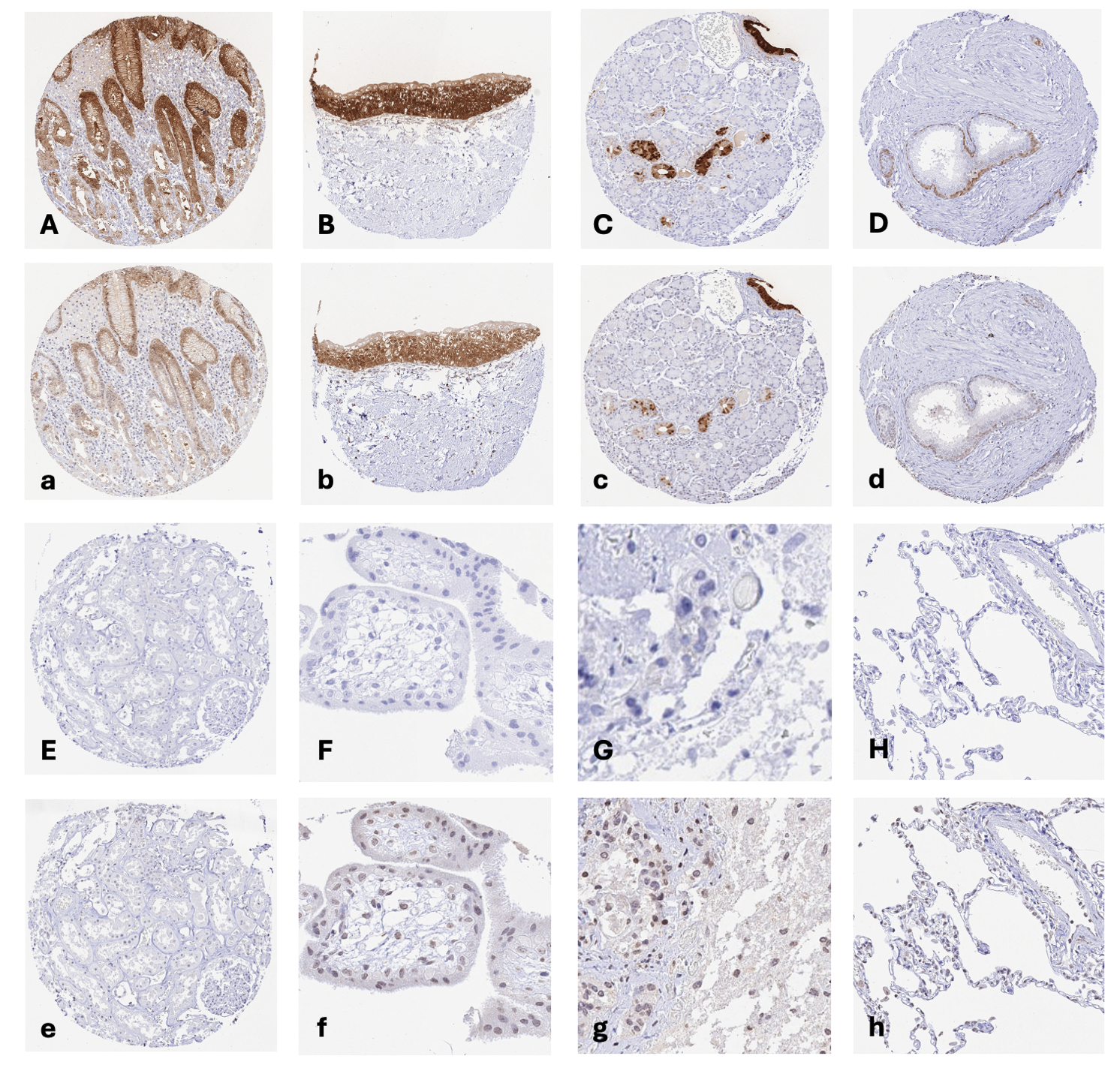


**Supplementary Figure 1.** **Assay validation by comparison of antibodies.** The panels show immunostaining results obtained by two independent GPX2 antibodies. Using HMV301, a predominantly cytoplasmic and nuclear staining was seen in gastric glandular cells (strongest in the apical layer) (A), urothelium with less intensity in umbrella cells (B), excretory ducts of salivary gland (C), basal cells of prostate epithelium (D) while staining was absent in kidney (E), placenta (F), neurohypophysis (G) and the lung (H). Using ab137431, a comparable staining was seen in the stomach (a), urothelium (b), salivary gland (c), prostate (d) and the kidney (e). A nuclear staining which was not seen by HMV301 was observed by ab137431 in trophoblasts of placenta (f), pituicytes of the neurohypophysis (g) and in lung pneumocytes (h). These stainings were considered an antibody specific cross-reactivity of ab13743. The images A-H and a-h are from consecutive tissue sections.

**Supplementary Figure 2. Examples of a positive and negative GPX2 immunostaining in normal tissues.** The panels show a moderate to strong GPX2 staining using the two independent antibodies HMV301 (A–H) and ab137431 (a–h) on consecutive tissue sections of the rectum (A), the gallbladder (B), the ileum (C) and the urothelium of the renal pelvis (D), whereas GPX2 staining is absent in the renal cortex (E), the thyroid gland (F), the placenta (G) and the tonsil (H).
